# Stubborn Swellings: A Rare Case of Kimura’s Disease Presenting as Parotid Swellings

**DOI:** 10.7759/cureus.59570

**Published:** 2024-05-03

**Authors:** Pranjal Rai, Sumankumar Ankathi, Nitin Panchal, Amit Janu, Subhash Yadav

**Affiliations:** 1 Radiodiagnosis, Tata Memorial Hospital, Homi Bhabha National Institute (HBNI), Mumbai, IND; 2 Pathology, Tata Memorial Hospital, Homi Bhabha National Institute (HBNI), Mumbai, IND

**Keywords:** angiolymphoid hyperplasia with eosinophilia, peripheral eosinophilia, cervical lymphadenopathy, radio-pathological correlation, head and neck swellings, kimura's disease

## Abstract

Kimura disease (KD) is a rare chronic inflammatory disorder characterized by the development of painless subcutaneous nodules, predominantly in the head and neck region. Diagnosis relies on a high index of clinical suspicion and clinicopathological correlation, with core biopsy serving as the gold standard for a definitive diagnosis. While the disease itself is benign, it can cause significant morbidity if left untreated. This case report describes a 48-year-old male who presented with bilateral infraauricular swellings, pruritus, and elevated serum IgE levels along with eosinophilia. Imaging and histopathological correlation confirmed the diagnosis of KD. Combination therapy of corticosteroids and cyclosporine resulted in significant clinical improvement, highlighting the efficacy of the approach while avoiding surgical resection. This case emphasizes the importance of radiologic-pathologic correlation along with the use of serology to effectively diagnose KD, even in atypical presentations.

## Introduction

Kimura disease (KD), also known as “eosinophilic hyperplastic lymphogranuloma,” is an uncommon benign chronic inflammatory condition [1]. The disease presents as multiple painless subcutaneous nodules, predominantly in the head and neck, with coexisting lymphadenopathy and salivary gland hyperplasia [2]. The causative agent of this disease remains unidentified. However, the presentation exhibits an endemic pattern within the Asian population, particularly affecting middle-aged males [3]. The other frequently involved anatomical sites include the groin, orbit, and eyelids. Pruritis may be a frequent accompanying complaint by the patient, with peripheral blood smears showing eosinophilia and serum demonstrating raised IgE levels in almost all cases [4].

Around 10-60% of patients may also have co-existing renal involvement [3]. Due to the potential for misdiagnosis with similar lesions, a high index of clinical suspicion coupled with radiologic-pathologic correlation is crucial for accurate diagnosis. While the disease exhibits a benign clinical course, its propensity for local recurrence necessitates a more aggressive, multimodal treatment approach to achieve optimal outcomes. A simple excision alone may be insufficient for definitive management [5]. Here, we report a case of Kimura’s disease in a middle-aged male, presenting as bilateral infraauricular swellings.

## Case presentation

A 48-year-old male presented with complaints of bilateral postauricular swelling for 13 years with pruritus, which had gradually increased over a period of two years. There was no family history of cancer or any known comorbidities. On clinical examination, the swellings were diffuse and non-tender, without any significant overlying erythema.

Preliminary serological tests and peripheral blood counts showed elevated IgE levels (7505 IU/ml) within the serum and peripheral eosinophilia (2.44 × 10^9^/L).

**Figure 1:**
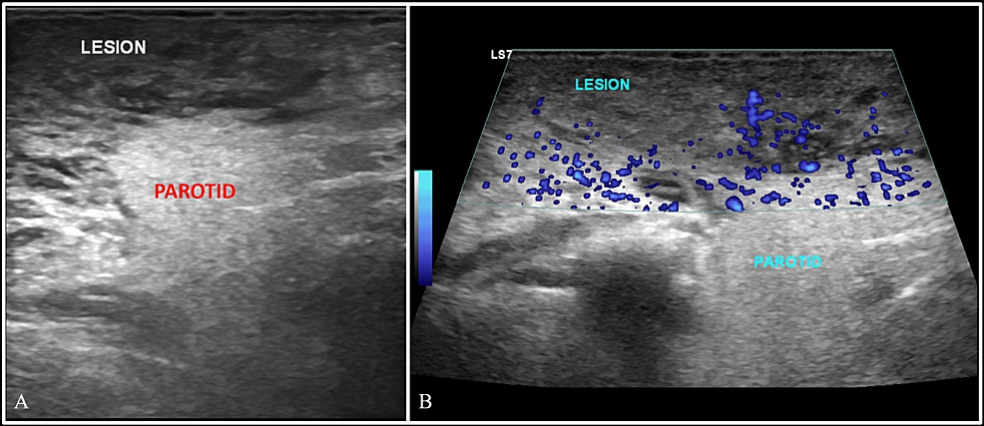
Greyscale ultrasound of the neck Greyscale ultrasound (A) and power doppler (B) images of the neck at the infraauricular level on the right side, showing a large heteroechoic mass superficial to the parotid gland, showing few areas of vascularity within it. Note the difference in the echotexture between the soft tissue mass and the parotid.

Initial imaging included a greyscale ultrasound of the neck, which revealed bilaterally symmetrical heteroechoic lesions in the retroauricular and infraauricular regions with a few scattered, enlarged cervical nodes at levels II, III, and IV on both sides. The lesions were superficial to the parotid glands and showed minimal vascularity within them (Figure 1).

**Figure 2:**
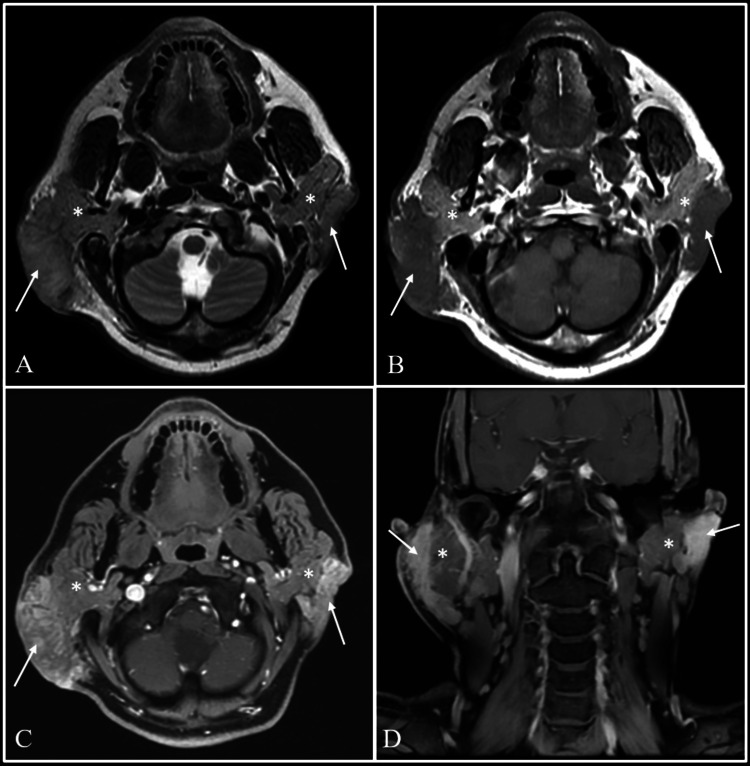
Evaluation with MRI Magnetic resonance imaging (MRI) with axial T2-weighted (A), axial T1-weighted (B), axial and coronal post-gadolinium T1-weighted images (C and D) showing T2 intermediate intensity lesions with heterogeneous post-contrast enhancement in bilateral infraauricular and retroauricular regions (arrows). The lesions are seen superficial to and separately from both the parotid glands, which is best appreciated on Pre-Gadolinium T1-weighted images (B). The parotid glands show normal signal intensity on both sides (asterisks).

Further evaluation with magnetic resonance imaging (MRI) was suggested, which demonstrated T2 isointense and short-tau inversion recovery (STIR) hyperintense lesions showing homogenous post-contrast enhancement in bilateral infraauricular and retroauricular regions, superficial to the parotid glands and seen separately from them, without any signal abnormality (Figure 2). A provisional diagnosis of KD was made based on the haemato-serological results and imaging findings.

**Figure 3:**
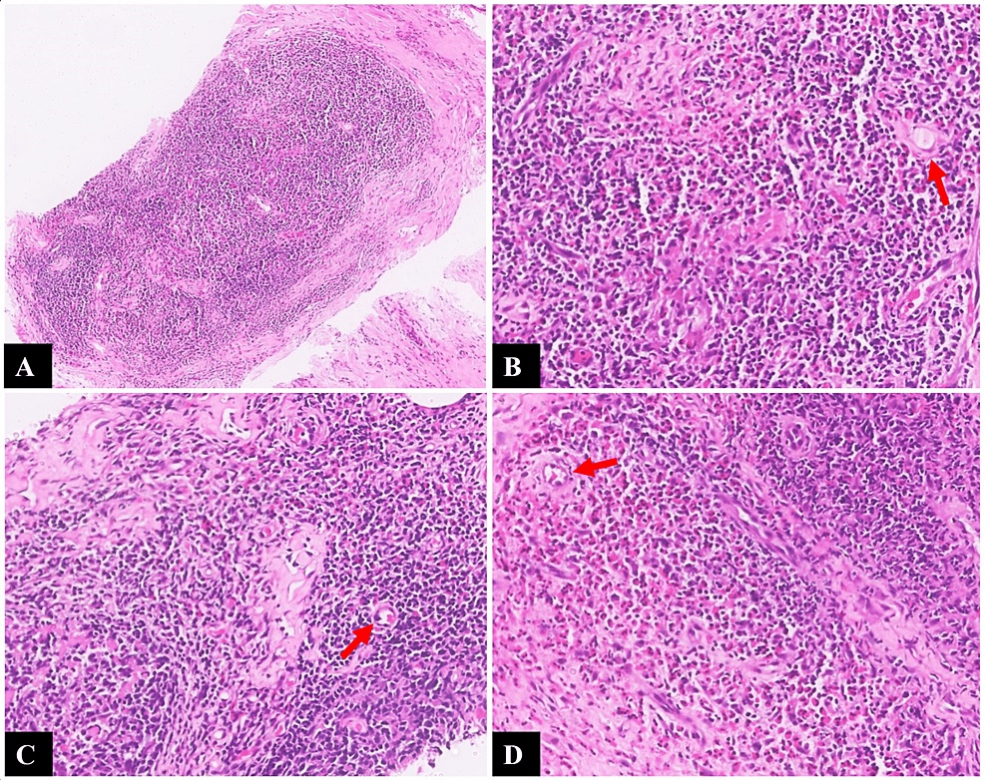
Histopathological findings (A) Nodal tissue with nodular architecture separated by dense fibrosis (×40, hematoxylin and eosin). (B-D) Higher-power images (×200, hematoxylin and eosin) showing sheets and lakes of eosinophilic infiltrates around the nodular architecture, with areas of capillary sized vascular proliferation and perivascular sclerosis (red arrows).

Fine-needle aspiration cytology from the right retroauricular region showed a hemorrhagic aspirate and a few lymphocytes, thus being non-contributory. This was followed by a core biopsy of the lesion. Histopathology revealed nodal tissue with nodular architecture separated by dense fibrosis along with an eosinophil-rich inflammatory infiltrate, thus confirming the diagnosis of KD (Figure 3).

Following the initiation of corticosteroid therapy (intravenous methylprednisolone followed by oral methylprednisolone), the patient experienced an improvement in symptoms and a decrease in the size of the bilateral supra-parotid masses after six days. In a collaborative decision following a multidisciplinary meeting, the patient's treatment plan was continued with combination immunosuppressive therapy. This regimen included low-dose corticosteroids and cyclosporin. Subsequent follow-up appointments at 6 and 12 months revealed a significant reduction in the size of the masses.

## Discussion

Kimura’s disease (KD) was first described by Chinese surgeons Kim and Szeto in 1937. However, its original histopathological features were described by Kimura et al. in 1948 [1]. The disease is endemic in the Asian demographic, with few sporadically appearing cases seen in the Western population. The male-to-female ratio is 4.5:1, and the median diagnostic age is 14 years [6]. It is noteworthy that our patient's age of 48 is above the median reported for Kimura disease diagnosis.

The usual presentation of KD is a triad of slow-growing swellings with a prolonged course, predominantly in the head and neck region, peripheral eosinophilia, and raised serum IgE levels [4]. This was evident in our case. 

The aetiology of KD remains elusive, with several potential triggers under investigation. One hypothesis proposes that an unidentified persistent antigenic stimulus may initiate a self-limited allergic or autoimmune response [7]. Infectious agents, including fungal or viral pathogens or even arthropod bites, have been implicated in potentially altering T-cell immunoglobulin function and triggering a type 1 hypersensitivity reaction [8]. Studies have demonstrated an association between the development of lymphoid follicles, elevated serum IgE levels, and the proliferation of Th2-type CD4+ T cells, leading to the overproduction of cytokines like granulocyte-macrophage colony-stimulating factor (GM-CSF), interleukins, and TNF-α. This suggests a potential role for clonal T-cell populations in disease development and recurrence [9]. This immune dysfunction can also predispose the patient to the development of allergic conditions such as pruritis, rhinitis, urticaria, and asthma [6]. Immunological involvement of the kidney can result in extra-membranous glomerulonephritis and nephrotic syndrome, manifesting as proteinuria [10]. Our case presented with pruritis without any involvement of the kidney or the presence of other allergic conditions.

Ultrasonography (US) plays a valuable role in the evaluation of head and neck lesions and lymph nodes, including guiding biopsy procedures. Sonographic features characteristically include enlarged, solid hypoechoic lymph nodes with preserved hilar architecture. Subcutaneous masses typically appear as hypoechoic or heteroechoic lesions, demonstrating mild internal vascularity [11].

MRI offers detailed visualization of well-defined lesions in the head and neck region. The signal intensity on MRI varies depending on the degree of vascularity and fibrosis within the lesion. Typically, lesions appear hypointense relative to normal salivary gland tissue on T1-weighted images and hyperintense on T2-weighted images. However, lesions with increased fibrosis may appear isointense or hypointense on T2-weighted images. Post-contrast T1-weighted sequences typically demonstrate intense homogeneous enhancement within the lesions [12].

Fine-needle aspiration cytology (FNAC) of the lesions reveals cellular smears composed of polymorphous population histiocytes, lymphoid cells, and endothelial cell clusters in a background of eosinophils, lymphohistiocytes, inflammatory cells, fibrous stroma, and haemorrhage [13]. When FNAC proves inconclusive, as in our case, a core biopsy serves as the gold standard for diagnosing KD. The most consistent histopathological features include the presence of aggregates of lymphoid follicles with germinal centre hyperplasia in the background of eosinophilic infiltration. Affected nodes demonstrate preservation of nodal architecture. Other frequently encountered findings of note include post-capillary venule proliferation, sclerosis, and the presence of proteinaceous deposits in the germinal centres [7]. FNAC carries a risk of false-negative results and non-diagnostic aspirates, which underscores the limitations of this technique, as seen in our case. Histopathological features in our case were largely concordant with the classic description of the disease.

Angiolymphoid hyperplasia with eosinophilia (ALHE) represents the closest differential diagnosis and was considered within the KD spectrum until recently (Table 1). However, several key features help distinguish these entities. ALHE exhibits a distinct demographic predilection, typically affecting middle-aged females in all racial groups. Clinically, ALHE patients are less likely to present with adenopathy, and serum IgE levels are often normal, in contrast to KD [5,14]. Other differentials for bilateral parotid/infraauricular masses, as seen in our case, would include lymphoma, salivary gland tumours, and Sjogren's syndrome [15]. Lymphomas are highly cellular tumours that will demonstrate diffusion restriction and intense homogenous post-contrast enhancement. Cross-sectional imaging not only showed the lesions in our case separately from parotid glands, but they also did not exhibit diffusion restriction.

**Table 1:** Distinguishing features of Kimura disease and angiolymphoid hyperplasia with eosinophilia Table Credits: Pranjal Rai AHLE: angiolymphoid hyperplasia with eosinophilia

Features	Kimura’s disease	ALHE
Demographic	Asian males	All racial groups with a female predominance
Presentation	Solitary subcutaneous lesions with pruritis, lymphadenopathy, and salivary gland involvement	Multiple, superficial, dermal erythematous nodules. No lymphadenopathy.
Pathophysiology	Chronic inflammatory disorder	Vascular proliferation secondary to inflammatory stimulation or damage
Systemic involvement	Salivary gland and renal involvement is more commonly seen	Rare
Histopathology	Follicular hyperplasia with maintained nodal architecture, separated with dense fibrosis and predominant eosinophilic infiltrate. IgE deposits may be present in the germinal center.	Vascular proliferation with endothelial aggregates and lobules lined by hobnail-shaped endothelial cells
Serological features	Peripheral eosinophilia with raised IgE levels	Normal laboratory investigations
Growth rate	Lesions grow more rapidly (1-4 years)	Slower growth pattern (up to 25 years)

Several published case reports describe parotid involvement in KD patients [15-22]. Some of these reports also mention concomitant lymph node involvement. Notably, in our case, although the infra-auricular masses mimicked parotid swelling, T1-weighted sequences clearly demonstrated their superficial location relative to the parotid glands on both sides. This distinguishes our case from previously reported instances of true intraparotid involvement.

Several treatment options exist for KD, including surgical excision, corticosteroids, immunosuppressive therapy, and radiation therapy. In cases of small tumours, surgical excision may be considered primarily for achieving cosmetic improvement. However, this approach is associated with a higher risk of recurrence, particularly for lesions with indistinct margins or larger sizes. Reported recurrence rates following surgical excision range from 30.5% to 100% [22,23]. Oral corticosteroids demonstrate efficacy in managing the disease; however, their use as monotherapy is often limited by post-withdrawal relapses. Combination therapies incorporating immunosuppressive agents, such as azathioprine, cyclophosphamide, cyclosporine, and mycophenolate mofetil, have shown improved clinical control and reduced recurrence rates [6]. Local radiation therapy can be opted for patients with positive surgical margins and repeated post-operative recurrences [23]. Our case demonstrates the benefits of combination therapy. This approach achieved excellent clinical improvement, obviating the need for surgical excision. Surgical intervention would have entailed the removal of multiple lesions, potentially leading to increased patient morbidity and a higher risk of recurrence.

KD generally carries a favourable prognosis. However, local recurrences can significantly impact patient morbidity if optimal treatment strategies are not implemented. Integrating imaging findings with serological evaluations for accurate diagnosis of this uncommon yet important disease entity can lead to the avoidance of invasive procedures like core-needle biopsies.

## Conclusions

KD remains a perplexing entity within the current clinical landscape, posing significant therapeutic challenges. A multimodality treatment approach guided by radiologic-pathologic correlation represents the current gold standard. This case report highlights the diagnostic challenges associated with KD in atypical presentations. Despite exceeding the median age of onset and presentation as bilateral infraauricular swellings, the patient's clinical course, serological profile, and imaging findings in our case were consistent with KD. The case emphasizes the importance of maintaining a broad differential diagnosis and integrating clinical features, laboratory findings, and radiologic investigations for an accurate diagnosis. Additionally, it underscores the efficacy of combination therapy in achieving significant clinical improvement and potentially avoiding the need for surgical intervention. Further research into the pathogenesis of KD is warranted to facilitate the development of targeted therapies and individualized management plans based on patient age and disease severity.
